# Differences in the Propensity of Different Antimicrobial Resistance Determinants to Be Disseminated via Transformation in *Campylobacter jejuni* and *Campylobacter coli*

**DOI:** 10.3390/microorganisms10061194

**Published:** 2022-06-10

**Authors:** Zahra Hanafy, Jason A. Osborne, William G. Miller, Craig T. Parker, Jonathan W. Olson, James H. Jackson, Sophia Kathariou

**Affiliations:** 1 Department of Food, Bioprocessing and Nutrition Sciences, North Carolina State University, Raleigh, NC 27695, USA; zehanafy@ncsu.edu (Z.H.); jhjackso@ncsu.edu (J.H.J.III); 2 Department of Statistics, College of Sciences, North Carolina State University, Raleigh, NC 27695, USA; jaosborn@ncsu.edu; 3 Produce Safety and Microbiology Research Unit, Agricultural Research Service, U.S. Department of Agriculture, Albany, CA 94710, USA; william.miller@usda.gov (W.G.M.); craig.parker@usda.gov (C.T.P.); 4 Department of Biological Sciences, North Carolina State University, Raleigh, NC 27695, USA; jonathan_olson@ncsu.edu

**Keywords:** antimicrobial resistance, AMR, *Campylobacter*, transformation

## Abstract

*Campylobacter jejuni* and *Campylobacter coli* are leading zoonotic foodborne pathogens, and the drugs of choice for human campylobacteriosis are macrolides (e.g., erythromycin) and fluoroquinolones. *C. jejuni* and *C. coli* are naturally competent for transformation via naked DNA uptake, but potential differences in transformation frequency (TF) for different antimicrobial resistance (AMR) markers remain poorly understood. We determined TFs for resistance to different antibiotics using as recipient a derivative of *C. jejuni* NCTC 11168 (strain SN:CM) with donor DNA from multidrug-resistant *C. jejuni* or *C. coli*. TF for nalidixic acid resistance ranked significantly highest (~1.4 × 10^−3^), followed by resistance to streptomycin and gentamicin. Tetracycline resistance via chromosomal *tet*(O) was less commonly transferred (~7.6 × 10^−7^), while transformation to erythromycin resistance was rare (≤4.7 × 10^−8^). We also determined TFs with the contemporary poultry-derived strains *C. jejuni* FSIS 11810577 and *C. coli* FSIS 1710488 as recipients. TFs to nalidixic acid and streptomycin resistance remained the highest (~7 × 10^−4^). However, TF for gentamicin resistance was remarkably low in certain recipient–donor combinations, while average TF for erythromycin resistance was noticeably higher (~3 × 10^−6^) than with SN:CM. Findings from this experimental model provide insights into factors that may impact transformation-mediated transfer of AMR leading to AMR dissemination in the agricultural ecosystem.

## 1. Introduction

*Campylobacter* spp. are spiral, obligately-microaerophilic zoonotic foodborne pathogens and are a leading bacterial cause of foodborne disease globally [1,2,3,4,5]. Recently, FoodNet listed *Campylobacter* as one of the leading agents for foodborne illness in the United States, with 19.5 cases per 100,000 population in 2019, an increase by 13% compared to 2016–2018 [4]. *Campylobacter* infects humans through direct contact with the feces of infected animals or via contaminated food and water, causing campylobacteriosis [3,4,5,6]. In addition to acute gastroenteritis, campylobacteriosis is a leading antecedent for the severe autoimmune complication Guillain–Barré Syndrome, which initially presents with weakness in the extremities and can progress to paralysis, as well as other sequelae [7,8]. Food animals such as poultry, cattle, swine, and sheep are major reservoirs for *Campylobacter* implicated in human illness, with poultry being a leading vehicle [5,9]. In the United States and other industrialized nations, most cases of human campylobacteriosis involve *Campylobacter jejuni,* followed by *C. coli* [1,10,11]. 

In the 2019 report on Antibiotic Resistance Threats by the Centers for Disease Control and Prevention (CDC), drug-resistant *Campylobacter* was listed among the “serious threats” to humans [12]. Human campylobacteriosis is typically self-limiting, but fluoroquinolones (e.g., ciprofloxacin, related to the quinolone antibiotic nalidixic acid) and macrolides (e.g., erythromycin and azithromycin) are commonly used to treat severe *Campylobacter* infections. Macrolides are increasingly employed due to the high prevalence of fluoroquinolone resistance in *C. jejuni*. Aminoglycosides (e.g., gentamicin) can also be employed to treat certain cases [13,14]. High prevalence of fluoroquinolone resistance in *Campylobacter* from human cases has prompted a ban of these antibiotics in poultry production [15]. 

*C. jejuni* and *C. coli* have been extensively documented to exhibit natural competence, and natural transformation is a major route for antimicrobial resistance (AMR) gene acquisition via horizontal gene transfer in *Campylobacter* [16,17]. Previous studies on transformation-mediated transfer of AMR traits in *C. jejuni* and *C. coli* have utilized various resistance markers (e.g., nalidixic acid, gentamicin, kanamycin, streptomycin, and erythromycin) [18,19,20,21,22]. In *C. coli*, transformation frequencies to nalidixic acid and erythromycin resistance were found to frequently differ, and to also be differentially impacted by temperature [18]. However, our understanding of potential differences in the transformation-mediated transfer of AMR determinants in *C. jejuni* and *C. coli* remains limited. In this work, we used a derivative of the extensively-employed reference strain *C. jejuni* NCTC 11168 [23,24] to determine the variation in transformation-mediated acquisition of different AMR phenotypes by this strain. Furthermore, trends in transformation-mediated acquisition of different resistance markers were investigated with contemporary strains of *C. jejuni* and *C. coli* isolated through government surveillance programs of poultry carcasses in the United States. 

## 2. Materials and Methods 

### 2.1. Bacterial Strains and Growth Conditions

The *Campylobacter* strains used in this study are listed in Table 1. Recipient strains were susceptible to the antimicrobials employed in the transformations and included *C. jejuni* Cj0264c::Cm (hereafter designated SN:CM), a derivative of *C. jejuni* NCTC 11168 that had been rendered resistant to chloramphenicol [25], *C. jejuni* FSIS 11810577, *C. coli* FSIS 1710488, and *C. coli* CVM N43850 (Table 1). *C. jejuni* SN:CM was originally chosen as recipient because we had deemed its chloramphenicol resistance marker desirable for potential use in future studies of transformations involving mixtures of live strains. Strains employed as sources of donor DNA for transformations were chosen based on their resistance to specific antimicrobials and were isolated by our laboratory during previous studies of *Campylobacter* in the commercial turkey industry in North Carolina, USA. Strains were routinely grown on Mueller-Hinton agar (MHA), consisting of Mueller-Hinton broth (MHB) (Becton, Dickinson and Co., Franklin Lakes, NJ, USA) with 1.2% agar at 42 °C under microaerobic conditions in a Bactrox Hypoxia Chamber (Shel Lab, Cornelius, OR, USA). Cultures were preserved at −80 °C in brain heart infusion broth (BHI; Becton, Dickinson and Co.) supplemented with 20% glycerol. 

### 2.2. DNA Isolation from Donor Strains

Bacteria were grown on MHA overnight (15–18 h) and a loopful of the culture was suspended in 600 μL of sterile dH_2_O. DNA isolation was performed using the QIAGEN DNeasy blood and tissue kit (QIAGEN, Germantown, MD, USA) following the vendor’s guidelines. Donor genomic DNA was suspended in 100 μL elution buffer and quantified using NanoDrop 2000 (Thermo Scientific, Waltham, MA, USA), with an average concentration of 177 ng/µL and a purity of 1.8–2 at A260/280. DNA was stored at −20 °C.

### 2.3. Transformation and Determination of Transformation and Mutation Frequencies

A loopful of an agar-grown culture was suspended in 1 mL MHB, and 25 µL of the cell suspension was spotted in duplicate onto MHA. Donor DNA (approx. 700 ng) was added to each spot and gently mixed using a micropipette tip. Spots were allowed to dry in a NuAire biosafety cabinet (Class 2, type A/B3) and plates were incubated overnight at 42 °C microaerobically. Each spot was then removed in its entirety using a sterile plastic loop and suspended in 500 µL MHB. Appropriate dilutions were spotted (10 µL, in duplicate) or spread-plated on MHA with appropriate antibiotics: tetracycline (4 µg/mL), gentamicin (4 µg/mL), kanamycin (64 µg/mL), nalidixic acid (32 µg/mL), streptomycin (64 µg/mL), and erythromycin (16 µg/mL). Plates were incubated at 42 °C microaerobically for 36–48 h before colonies were enumerated. Total CFUs of the recipient at the end of the transformation period were enumerated by plating appropriate dilutions on MHA without any antibiotics. Transformation frequency was calculated as the ratio of total CFUs on MHA with the appropriate antibiotic over the total CFUs on MHA without any antibiotics. Cell suspensions from spots without added DNA were similarly diluted and plated on antibiotic-amended media to determine the frequency of spontaneous resistance mutations for resistance to the corresponding antibiotic. Mutation frequency was calculated as the ratio of total CFUs in the control spot without added DNA at the end of the incubation period on MHA supplemented with the appropriate antibiotic over the total CFUs on MHA without any antibiotics. Single colonies of representative transformants or spontaneous mutants were subcultured on MHA with the respective antibiotic and preserved at −80 °C, as described above. 

### 2.4. Minimum Inhibitory Concentration (MIC) Determinations

For certain transformants, the erythromycin MIC was determined using Liofilchem^®^ MIC Test Strips (Waltham, MA, USA) with antibiotic concentrations ranging from 0.016 to 256 μg/mL, following the vendor’s guidelines. Plates were incubated at 42 °C microaerobically for 36–48 h and the MIC was measured by observing the lowest concentration where growth was inhibited.

### 2.5. Confirmation of Resistance Determinants

PCR was used to confirm presence of specific resistance determinants in transformants. Transformants selected on gentamicin or kanamycin were tested for the presence of *aph(2”)-If* (chromosomally borne in the donor strains utilized here) using primers 5′ AAG GAA CTT TTT TAA CAC CAG 3′ and 5′ CCW ATT TCT TCT TCA CTA TCT TC 3′ [30], as well as for the plasmid-borne *aphA-3* using primers 5′ GAAAGCTGCCTGTTCCAAAG 3′ and 5′ ATGTTGCTGTCTCCCAGGTC 3′ [26]. Tetracycline-resistant transformants were tested for *tet*(O) using primers 5′ CAA AGG GGA ATC ACT ATC C 3′ and 5′AAC CTG CCC GCA TAG TTC 3′ [29]. 

### 2.6. Bioinformatics Analysis of Gentamicin Resistance Regions in Donor and Recipient Genomes

Chromosomal DNA sequences that flank the gentamicin resistance-encoding IS*1595* element were extracted from the donor strain genome sequences (Table 1). The corresponding regions were also extracted from the recipient strain genomes (Table 1), with the sequence of *C. jejuni* 11688 being used for *C. jejuni* SN:CM in the analyses. The flanking sequences for donor strains *C. jejuni* 14980A, *C. coli* 14983A, and *C. jejuni* 15065A spanned the chromosomal regions *murD-accA*, *Cj1477c-addB,* and *umpG-bioC*, respectively. Pairwise BLASTN comparisons were performed for each recipient strain against each donor strain. For each pair, the BLASTN output and the cognate donor and recipient sequences were imported into the Artemis Comparison Tool (ACT) ver. 18.1 [31]. For each comparison, the minimum percent ID cutoff was set to 75. Pairwise comparisons are illustrated in Supplementary Figures S1–S3.

### 2.7. Statistical Analysis

To assess the statistical significance and differences in transformation frequencies for different antibiotic resistance markers in each recipient strain, the Tukey–Kramer test was used with SAS software (SAS Institute, Cary, NC, USA). A reduced model was considered with factorial effects that accommodated different means for combinations of donor species (i.e., *C. coli* or *C. jejuni*), recipient strains (i.e., SN:CM, FSIS 11810577 or FSIS 1710488), and resistance markers (erythromycin, gentamicin, kanamycin, nalidixic acid, streptomycin, and tetracycline). The effects of donor strain were modeled with random effects by pooling all effects involving the donor strain into a single term. The model *Y_ijklmn_ = μ_ijk_ + D_l(ijk)_ + B_m(ijkl)_* was used where the last two terms denoted with upper case letters are random effects for donor trial (D) and biological replicates (B); *i*, *j*, *k*, *l*, *m,* and *n* denote donor strain, donor species, recipient strain, resistance marker, trial, and number of replicates in each trial, respectively.

## 3. Results and Discussion 

### 3.1. Differences in Transformation Frequencies of C. jejuni SN:CM for Different Resistance Markers 

A first series of transformations employed as recipient the reference strain *C. jejuni* SN:CM (Cm^R^) [25] and donor DNA from two sequenced multidrug-resistant strains, *C. jejuni* 14980A and *C. coli* 14983A, obtained from turkey feces and from a housefly (*Musca domestica*) in a turkey farm, respectively [27]. *C. jejuni* 14980A harbored plasmid-borne tetracycline (T) and kanamycin (K) resistance determinants as well as chromosomal determinants conferring resistance to streptomycin (S), kanamycin (K), nalidixic acid (Q), and gentamicin (G), with its AMR phenotype designated TSKQG [27]. *C. coli* 14983A harbored all the above determinants as well as a specific A:G transition in the three 23S rRNA genes, conferring resistance to erythromycin (E), with its AMR phenotype designated TSEKQG [27]. 

The observed transformation frequencies of *C. jejuni* SN:CM to a specific resistance were noticeably higher than the mutation frequencies to the corresponding antimicrobial. For instance, the average mutation frequencies to nalidixic acid resistance were 1.4 × 10^−7^ ± 2.5 × 10^−7^, while the average gentamicin and streptomycin resistance mutation frequencies were 3.5 × 10^−8^ ± 9.2 × 10^−8^ and <1.7 × 10^−9^ ± 5.9 × 10^−9^, respectively. Spontaneous mutants with resistance to erythromycin or tetracycline were extremely uncommon (<3.07 × 10^−9^ ± 2 × 10^−9^ and <8.4 × 10^−10^ ± 2 × 10^−9^, respectively). Thus, we considered the effect of spontaneous mutations to the specific antimicrobial resistance phenotypes investigated here low enough to be negligible in the calculations of transformation frequencies.

**Figure 1:**
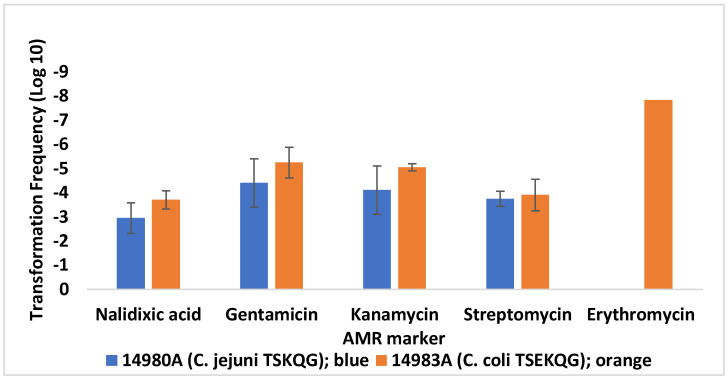
Transformation of *C. jejuni* SN:CM with DNA from *C. jejuni* 14980A and *C. coli* 14983A. Transformation frequencies are averages from two or more independent trials for each recipient strain–donor DNA pair. Letters within each AMR profile indicate resistance to streptomycin (S), gentamicin (G), kanamycin (K), erythromycin (E), or the quinolones nalidixic acid and ciprofloxacin (Q). No erythromycin-resistant transformants were obtained with DNA from *C. coli* 14983A (*C. jejuni* 14980A is susceptible to erythromycin, hence was not used as source of donor DNA), and the transformation frequencies are below the limit of detection (<10^−8^). The error bar on the column with the transformation frequency data for erythromycin resistance was too small to be visible. Transformations were carried out as described in Materials and Methods.

Highest transformation frequencies (TFs) were seen for transformation to nalidixic acid and streptomycin resistance (10^−4^–10^−3^), followed by gentamicin and kanamycin resistance. Significantly lower TFs (*p* < 0.05) were observed with erythromycin resistance (<10^−8^) (Figure 1). For each donor–recipient combination, the TFs to gentamicin resistance were similar overall to those for kanamycin resistance (Figure 1), in support of previous evidence that the gentamicin resistance gene *aph(2”)-If* also mediated resistance to kanamycin [32]. Gentamicin-selected transformants grew readily on MHA with kanamycin (64 µg/mL), and vice versa, kanamycin-selected transformants grew on MHA with gentamicin (4 µg/mL). Transformants selected on either gentamicin or kanamycin were confirmed via PCR to harbor *aph(2”)-If*, harbored chromosomally by the donor strains. Even though the donor strains also harbor plasmid-borne genes such as *aphA-3* [27,30] conferring resistance to kanamycin but not gentamicin, we confirmed via PCR that these genes were not transferred. This is to be expected since plasmid-borne genes fail to be transferred efficiently via transformation in *Campylobacter* [17,33]. 

**Figure 2:**
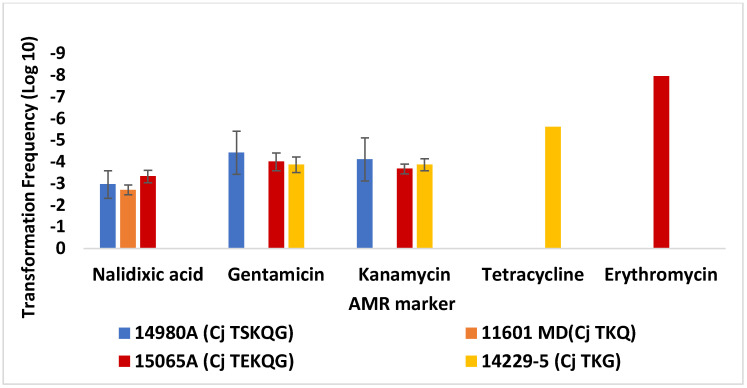
Transformation of *C. jejuni* SN:CM with DNA from a panel of *C. jejuni strains.* Transformation frequencies are averages from two or more independent trials for each recipient strain–donor DNA pair. Tetracycline resistance transformations were pursued only with DNA from *C. jejuni* 14229-5, which harbors a chromosomal tetracycline resistance gene [28]. Transformations to gentamicin and kanamycin resistance were performed with DNA from *C. jejuni* 14980A, 14229-5, and 15065A, all of which harbored a chromosomal *aph(2*′′*)-If*, while transformations to tetracycline resistance were performed with *C. jejuni* 14229-5, the only strain among these donors with a chromosomal *tet*(O). The data from transformations with *C. jejuni* 14980A shown in Figure 1 are included for comparison. Cj and Cc in front of strain designations indicate *C. jejuni* and *C. coli*, respectively. Letters within each AMR profile indicate resistance to tetracycline (T), gentamicin (G), kanamycin (K), erythromycin (E), or the quinolones nalidixic acid and ciprofloxacin (Q). The error bars on the columns with the transformation frequency data for tetracycline and erythromycin resistance were too small to be visible. Transformations were carried out as described in Materials and Methods.

Transformation of *C. jejuni* SN:CM using DNA from several other *C. jejuni* and *C. coli* strains yielded similar findings. Using DNA from these new *C. jejuni* donors, transformation of *C. jejuni* SN:CM to nalidixic acid resistance was again most frequently encountered, while transformation to erythromycin resistance was significantly less common, even when the erythromycin-resistant strain *C. jejuni* 15065A served as source for the donor DNA (Figure 2). The failure to obtain erythromycin-resistant transformants of *C. jejuni* SN:CM using DNA from *C. jejuni* 15065A (TF < 1.7 × 10^−8^) is noteworthy considering that nalidixic acid and gentamicin-resistant transformants could be readily obtained in the same transformation experiments, with the same donor DNA (average TF 5.6 × 10^−4^ and 4.5 × 10^−5^, respectively) (Figure 2). As observed earlier (Figure 1), TFs to gentamicin resistance were similar to those to kanamycin resistance (Figure 2).

**Figure 3:**
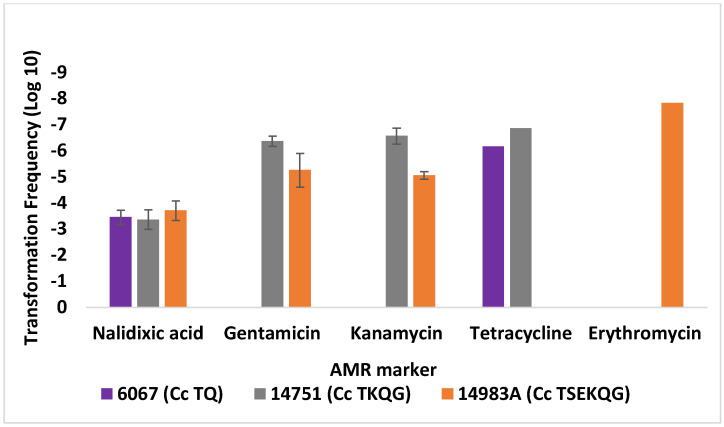
Transformation of *C. jejuni* SN:CM with DNA from a panel of *C. coli* donors. Transformation frequencies are averages from two or more independent trials for each recipient strain–donor DNA pair, and transformations were carried out as described in Materials and Methods. The data from transformations with *C. coli* 14983A shown in Figure 1 are included for comparisons. Cj and Cc in front of strain designations indicate *C. jejuni* and *C. coli*, respectively. AMR phenotype designations in strain names are as in the legend to Figure 2. The error bars on the columns with the transformation frequency data for tetracycline and erythromycin resistance were too small to be visible.

Similar findings were obtained in transformations of *C. jejuni* SN:CM using DNA from additional *C. coli* strains (Figure 3). Generally, TFs were higher intra-specifically than inter-specifically (*p* < 0.05), as has been previously reported [17,34]. Transformation to nalidixic acid, gentamicin, kanamycin, and streptomycin resistance was significantly more common than to erythromycin resistance (Figure 3). As noted earlier, TFs to gentamicin and kanamycin resistance were similar for a specific donor–recipient combination (Figure 3). Collectively, the data indicated that transformation frequency of *C. jejuni* SN:CM to erythromycin resistance was significantly lower than to all other tested markers, regardless of the source of donor DNA, with the difference being especially notable when compared to the transformation frequencies for nalidixic acid or gentamicin resistance (*p* < 0.0001).

*C. jejuni* 14980A (TSKQG) and *C. coli* 14983A (TSEKQG) harbored plasmid-borne *tet*(O), conferring resistance to tetracycline [27]. We failed to detect acquisition of tetracycline resistance by *C. jejuni* SN:CM via transformation using DNA from these strains (TF < 10^−8^), in agreement with previous evidence that transformation-mediated acquisition of plasmid-borne genes is uncommon in *Campylobacter*, as also mentioned above [17,33]. Therefore, tetracycline resistance transformation frequencies for SN:CM were assessed using as source of donor DNA the strain *C. coli* 6067 (TQ), harboring a chromosomal *tet*(O) [29], as well as *C. jejuni* 14229-5 (TKG), which also harbors a chromosomal *tet*(O) [28] and *C. coli* 14751 (TKQG), which was known from previous pilot studies to be able to serve as donor for tetracycline resistance determinations (Z. Hanafy and S. Kathariou, unpublished findings) and likely harbors a chromosomal *tet*(O). The average transformation frequencies to tetracycline resistance using DNA from these strains ranged from ~2 × 10^−7^ to ~3 × 10^−6^ (Figure 2 and Figure 3), and all tested tetracycline-resistant transformants were found via PCR to harbor *tet*(O). Thus, transformation frequency of *jejuni* SN:CM to tetracycline resistance using donors with chromosomal *tet*(O) was lower than to nalidixic acid or streptomycin resistance (*p* < 0.005), and, depending on the donor, it was either similar or lower than to gentamicin or kanamycin resistance, but still higher than observed for transformation to erythromycin resistance (Figure 3).

### 3.2. Contemporary Industry-Relevant Campylobacter Strains Exhibit Variable Outcomes for Transformation to Gentamicin Resistance

*C. jejuni* SN:CM is derived from the type strain *C. jejuni* NCTC 11168 deposited at the National Collection of Type Cultures in 1977, and has been extensively propagated and investigated in various laboratories [35]. To determine whether the data with *C. jejuni* SN:CM showed similar patterns to *Campylobacter* strains of greater current relevance for the poultry industry, we used as recipients the pan-sensitive strains *C. jejuni* FSIS 11810577 and *C. coli* FSIS 1710488, isolated in 2018 during USDA-FSIS surveillance of chicken carcasses in the United States, as well as *C. coli* N43850, isolated from chicken breast in 2013 and exhibiting resistance to tetracycline, gentamicin, and kanamycin (Table 1). These strains were found in previous assays to be competent for transformation (Z. Hanafy, M. Miller, and S. Kathariou, unpublished findings). Their spontaneous mutation frequencies to the different resistance phenotypes exhibited the same low values described above for *C. jejuni* SN:CM, and thus their contribution to the calculations of transformation frequencies was considered to be negligible.

**Figure 4:**
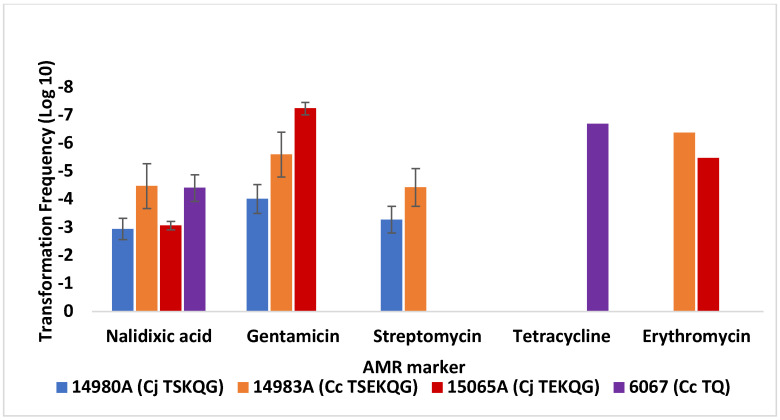
Transformation of *C. jejuni* FSIS 18110577 with DNA from a panel of *C. jejuni* and *C. coli* donors. Transformation frequencies are averages from two or more independent trials for each recipient strain–donor DNA pair, and transformations were carried out as described in Materials and Methods. Cj and Cc in front of strain designations indicate *C. jejuni* and *C. coli*, respectively. AMR phenotype designations in strain names are as in the legend to Figure 2. The error bars on the columns with the transformation frequency data for tetracycline and erythromycin resistance were too small to be visible.

**Figure 5:**
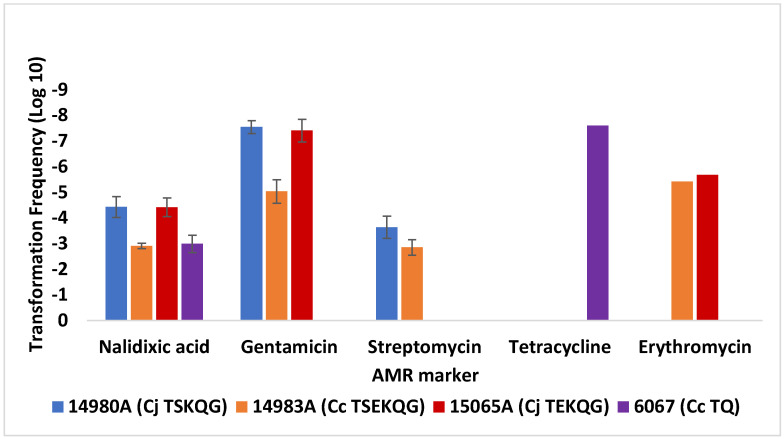
Transformation of *C. coli* FSIS 1710488 to different AMR markers with DNA from a panel of *C. jejuni* and *C. coli* strains. Transformation frequencies are averages from two or more independent trials for each recipient strain–donor DNA pair, and transformations were carried out as described in Materials and Methods. Cj and Cc in front of strain designations indicate *C. jejuni* and *C. coli*, respectively. AMR phenotype designations in strain names are as in the legend to Figure 2. The error bars on the columns with the transformation frequency data for tetracycline and erythromycin resistance were too small to be visible.

Both *C. jejuni* FSIS 11810577 and *C. coli* FSIS 1710488 readily yielded transformants to nalidixic acid and streptomycin resistance, and frequencies of transformation to these resistance traits were significantly higher than observed for other markers such as gentamicin, tetracycline, or erythromycin (*p* < 0.05) (Figure 4 and Figure 5). Furthermore, as discussed above with *C. jejuni* SN:CM, transformation frequencies for resistance to gentamicin were similar to those for resistance to kanamycin. As with *C. jejuni* SN:CM, transformants selected on either gentamicin or kanamycin were confirmed via PCR to harbor *aph(2″)*-*If*, harbored in the chromosome of *C. jejuni* 14980A used as source of donor DNA, but not *aphA-3*, which is plasmid-borne in this donor strain [27]. However, unlike *C. jejuni* SN:CM, gentamicin resistance transformation frequencies showed surprisingly high dependence on the source of the donor DNA. Specifically, while *C. jejuni* FSIS 11810577 could be readily transformed to gentamicin resistance using DNA from either *C. jejuni* 14980A or *C. coli* 14983A, it exhibited much lower transformation frequency to gentamicin resistance with DNA from *C. jejuni* 15065A (Figure 4). In contrast, *C. jejuni* SN:CM had readily yielded gentamicin-resistant transformants with DNA from *C. jejuni* 15065A (Figure 2). Furthermore, *C. coli* FSIS 1710488 failed to be readily transformed to gentamicin resistance using DNA from either *C. jejuni* 15065A or *C. jejuni* 14980A (Figure 5). Gentamicin resistance TFs of *C. coli* FSIS 1710488 were significantly lower than those of *C. jejuni* SN:CM or *C. jejuni* FSIS 11810577 with the same donor DNA, e.g., DNA from *C. jejuni* 14980A (*p* < 0.0001) (Figure 1 and Figure 4). This failure to transform appeared specific to gentamicin, since other markers (e.g., nalidixic acid or streptomycin resistance) could be readily acquired via transformation in the same recipient–donor combinations (Figure 4 and Figure 5).

Gentamicin resistance mediated by *aph(2”)-If* can be acquired by *C. jejuni* NCTC11688 via transformation with DNA from various *aph(2”)-If*-harboring *C. jejuni* or *C. coli* strains [36,37,38], but findings from diverse recipient–donor combinations have been largely lacking. Pairwise analysis of the nine donor–recipient combinations suggests that TFs are generally higher when the combinations are within the same species (e.g., *C. jejuni* NCTC 11168 recipient with DNA from *C. jejuni* 15065A), and that TF is largely driven by the DNA similarity between the regions that flank the gentamicin resistance-encoding IS*1595* element (Supplementary Figures S1–S3). This is not unexpected, as TF values would be predicted to decrease with increasing taxonomic distance and concomitant reductions in overall DNA similarity. However, blocks of increased or reduced homology within the flanking regions could impact the transformation frequency. The strongly conserved ribosomal genes *rpsI* and *rplM* flank the insertion site of the gentamicin resistance cassette in *C. coli* 14983A at the 5′ side (Supplementary Figure S1). This high homology, even within inter-specific combinations, could have resulted in the higher-than-expected TFs with *C. coli* 14983A donor DNA. Additionally, as stated above, TF of *C. jejuni* FSIS 11810577 by *C. jejuni* 15065A DNA was very low, even though this was an intra-specific combination. However, analysis of this combination indicates a decreased level of similarity within the 3′ flanking region (Supplementary Figure S3), when compared to another intraspecific pair (*C. jejuni* NCTC 11168 vs. *C. jejuni* 15065A). Thus, this lower level of similarity in the 3′ flanking region is one likely cause of the reduced transformation efficiency in this intra-specific combination. Finally, although not examined in this study, other factors, such as the distance between the flanking regions, may play a role in determining transformation efficiency and should be examined further.

### 3.3. Transformation Frequency of C. jejuni SN:CM to Erythromycin Resistance Is Markedly Lower Than Observed with Contemporary Campylobacter Strains from Poultry

**Figure 6:**
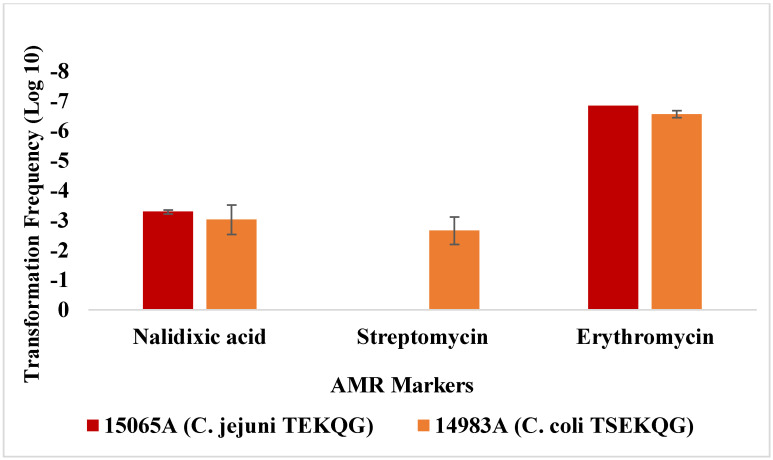
Transformation of *C. coli* N43850 to nalidixic acid, streptomycin, and erythromycin resistance. Transformation frequencies are averages from two or more independent trials for each recipient strain–donor DNA pair, and transformations were carried out as described in Materials and Methods. The error bar for transformation frequency to erythromycin resistance using DNA from *C. jejuni* 15065A was too small to be visible. Cj and Cc in front of strain designations indicate *C. jejuni* and *C. coli*, respectively. AMR phenotype designations in strain names are as in the legend to Figure 2.

As noted above, the frequency of transformation of *C. jejuni* FSIS 118110577 and *C. coli* FSIS 1710488 to erythromycin resistance was lower than for resistance to nalidixic acid or streptomycin (Figure 4 and Figure 5). However, it is noteworthy that erythromycin resistance transformation was overall significantly more common in these two strains than in *C. jejuni* SN:CM (*p* < 0.05) (Figure 4 and Figure 5). Similar findings were obtained with *C. coli* N43850, which could be transformed to nalidixic acid and streptomycin resistance readily, but for which erythromycin-resistant transformants could still be obtained, albeit at significantly lower frequency (Figure 6). This strain was not tested for transformation to gentamicin or kanamycin, since it harbors *aph(2”)-If* and is therefore already resistant to these antibiotics (Table 1).

The findings with the *C. coli* strains FSIS 1710488 and N43850 are similar to those previously reported with *C. coli* strains from turkeys or swine, for which transformation frequency to erythromycin resistance typically ranged between 10^−5^ and 10^−7^, with strains from swine exhibiting lower values within this range [18,19]. Frequencies of transformation of turkey-derived strains to nalidixic acid resistance were generally higher than for resistance to erythromycin [18], similarly to what was noted in the current study. This likely reflects the fact that transformation to high levels of erythromycin resistance requires transfer of all three 23S rRNA genes harboring the erythromycin resistance-associated substitutions, whereas nalidixic-acid-resistant transformants only need to acquire a single gene, *gyrA*, harboring the substitution associated with (fluoro)quinolone resistance [13,39,40].

The underlying reasons for the pronounced difficulty of *C. jejuni* SN:CM to be transformed to erythromycin resistance are unknown but may reflect high fitness costs of erythromycin resistance in this strain. Substantial evidence indicates that resistance to erythromycin and other macrolides is accompanied by significant fitness costs for colonization of birds in *C. jejuni* but, interestingly, not in *C. coli*, potentially accounting for the much higher prevalence of erythromycin resistance in the latter [13]. Colony size of erythromycin-resistant transformants of *C. jejuni* FSIS 118110577 was consistently smaller than with transformants to nalidixic acid resistance, while such an impact on colony size was not noted with *C. coli* FSIS 1710488. Upon subculture, some colonies of the erythromycin-resistant transformants of *C. jejuni* FSIS 118110577 remained small while others had normal size. Determination of the erythromycin MIC of putative transformants with small and normal-size colonies revealed high levels of resistance with MIC ≥ 256 μg/mL, regardless of colony size. It is thus tempting to speculate that even though erythromycin resistance confers substantial fitness costs to *C. jejuni*, some strains, such as FSIS 118110577, are much better able to cope with such costs, potentially via compensatory mutations, than others such as SN:CM. Even though the contemporary *C. jejuni* FSIS 118110577 strain was able to yield erythromycin-resistant transformants fairly effectively under laboratory conditions, the small colony size of the transformants still reflects accompanying fitness costs. On the other hand, transformation to erythromycin resistance was clearly impaired in *C. jejuni* SN:CM, and thus this strain may better serve as indicator for field outcomes of transformation-mediated erythromycin resistance dissemination in *C. jejuni* in the agricultural ecosystem.

## 4. Conclusions and Further Research

Different transformation frequency patterns were observed in this study, with several antimicrobial resistance markers among the tested recipients. Transformation frequencies to nalidixic acid and streptomycin resistance were consistently the highest, while erythromycin resistance transformation frequencies were noticeably lower, especially for *C. jejuni* SN:CM. Interestingly, in contemporary strains of *C. jejuni* and *C. coli* from the poultry industry transformation-mediated acquisition of erythromycin resistance was consistently higher than noted with *C. jejuni* SN:CM, possibly reflecting mitigation of the fitness costs of erythromycin resistance in transformants of these strains. For *C. jejuni*, such mitigation of fitness costs may be more likely under laboratory conditions, as erythromycin resistance remains overall much less common in this species than in *C. coli*. Another unexpected outcome of the use of the contemporary strains was the discovery that transformation-mediated transfer of gentamicin resistance via *aph(2”)-If* exhibited a pronounced dependence on the specific recipient–donor combination, which was not noted with transfer of other resistance markers such as nalidixic acid or streptomycin resistance. Further studies are needed to fully elucidate the impact of the genomic landscape of the *aph(2”)-If*-harboring islands on transformation outcomes. The genomes of transformants obtained in this study are currently being sequenced, and their analysis is expected to yield valuable insights on mechanisms such as co-transformation events and compensatory mutations that may impact the emergence and persistence of transformation-acquired antimicrobial resistance.

The experimental model employed in the current study utilized laboratory conditions and exposure of live cells to purified total genomic DNA. It will be of interest to determine whether similar findings can be obtained with alternative, more complex models such as those employing animal hosts inoculated with mixtures of cultures that may act as donors and recipients in transformation [28]. Furthermore, transformation in *C. jejuni* can take place under growth-restrictive conditions [18,20]. Further studies are needed to determine whether transformation of *C. jejuni* to certain resistance traits primarily takes place under conditions prevailing in animal hosts while other traits may be acquired efficiently via transformation both within and outside the animal host, as has been suggested by previous work with *C. coli* [18]. Such findings will be critical in elucidating antimicrobial resistance emergence and dissemination in *Campylobacter* in the food chain.

## Figures and Tables

**Table 1:** *Campylobacter* strains used in this study.

Strain ^1^	Source [Reference] ^2^	AMR Profile ^3^	Use	Genome Accession ^4^
*C. jejuni*				
SN:CM	NCTC 11168 derivative, strain Cj0264c::Cm [25]	Cm	Recipient	AL111168
11601MD	Jejunum, turkey, 2006 [26]	TKQ	Donor	LKCR00000000
14980A	Feces, turkey, 2014 [27]	TSKQG	Donor	CP017029
14229-5	Feces, turkey, 2013 [28]	TKG	Donor	https://www.ebi.ac.uk/biosamples/samples/SAMEA7848382 (accessed on 17 May 2022)
15065A	Cecum, turkey, 2014	TEKQG	Donor	CP092017
FSIS 11810577	Young chicken, 2018	Pan-sensitive	Recipient	AACOOF000000000
*C. coli*				
6067	Water, turkey house, 2003 [29]	TQ	Donor	LKCQ00000000
14983A	Housefly (*Musca domestica*) from turkey farm, 2014 [27]	TSEKQG	Donor	CP017025
14751	Cecum, turkey, 2013	TKQG	Donor	N/A
FSIS 1710488	Young chicken, 2017	Pan-sensitive	Recipient	AACKVZ000000000
CVM N43850	Chicken breast, 2013	TKG	Recipient	Biosample SAMN03344552; Assembly GCA_001417185.1

^1^ Strains *C. jejuni* FSIS 11810577 and *C. coli* FSIS 1710488 were kindly provided by Drs. John Johnston, Mustafa Simmons, and Glenn Tillman, USDA-ARS, Georgia, USA; *C. coli* CVM N43850 was kindly provided by Drs. Patrick McDermott and Shaohua Zhao, FDA. ^2^ NCTC, National Collection of Type Cultures. ^3^ AMR profile acronyms indicate resistance to tested antimicrobials i.e., tetracycline (T), streptomycin (S), gentamicin (G), kanamycin (K), erythromycin (E), nalidixic acid (Q), and chloramphenicol (Cm). A combination of the letters indicates a specific AMR profile; e.g., TQ refers to resistance to tetracycline and nalidixic acid but not to any of the other tested antimicrobials. Pan-sensitive indicates lack of resistance to any of the tested antimicrobials. ^4^ For strain SN:CM, the genome sequence of the parental strain NCTC 11168 was used in the genomic analyses; N/A, not available.

## Data Availability

All the data in this manuscript were in the Supplementary Materials.

## Supplementary Materials

The following supporting information can be downloaded at: https://www.mdpi.com/article/10.3390/microorganisms10061194/s1, Figure S1. Context/similarity comparisons between the genomic regions flanking the gentamicin resistance-encoding element in the donor strain *C. coli* 14983A and the corresponding regions in the three recipient strains *C. jejuni* NCTC 11168, *C. jejuni* FSIS 11810577 and *C. coli* FSIS 1710488. The IS*1595* and IS*605* elements are colored light blue and medium blue, respectively, with their cognate transposase genes colored magenta and violet, respectively. The *aph(2”)-**If* gentamicin resistance gene is in green. Pseudogenes are yellow. Average transformation frequencies for each pair are shown in blue font on the right. Regions of similarity between each genome pair are colored in shades of red that represent % nucleotide similarity, as determined through BLASTN analysis of the donor against each recipient. Figure S2. Context/similarity comparisons between the genomic regions flanking the gentamicin resistance-encoding element in the donor strain *C. jejuni* 14980A and the corresponding regions in the three recipient strains *C. jejuni* NCTC 11168, *C. jejuni* FSIS 11810577 and *C. coli* FSIS 1710488. The IS*1595* element is colored light blue with its cognate transposase gene colored magenta. The *aph(2”)-**If* gentamicin resistance gene is in green. Pseudogenes are yellow. Average transformation frequencies for each pair are shown in blue font on the right. Regions of similarity between each genome pair are colored in shades of red that represent % nucleotide similarity, as determined through BLASTN analysis of the donor against each recipient. Figure S3. Context/similarity comparisons between the genomic regions flanking the gentamicin resistance-encoding element in the donor strain *C. jejuni* 15065A and the corresponding regions in the three recipient strains *C. jejuni* NCTC 11168, *C. jejuni* FSIS 11810577 and *C. coli* FSIS 1710488. The IS*1595* element is colored light blue with its cognate transposase gene colored magenta. The *aph(2”)-**If* gentamicin resistance gene is in green. Average transformation frequencies for each pair are shown in blue font on the right. Regions of similarity between each genome pair are colored in shades of red that represent % nucleotide similarity, as determined through BLASTN analysis of the donor against each recipient.
