# Supplementary material for: Differences in the Propensity of Different Antimicrobial Resistance Determinants to Be Disseminated via Transformation in Campylobacter jejuni and Campylobacter coli
Source: Microorganisms. 2022 Jun 10;10(6):1194. doi: 10.3390/microorganisms10061194 (PMC9227638; doi:10.3390/microorganisms10061194)
Supplement: Supplementary file 1 [file microorganisms-10-01194-s001.zip › microorganisms-1755031-supplementary.pdf]

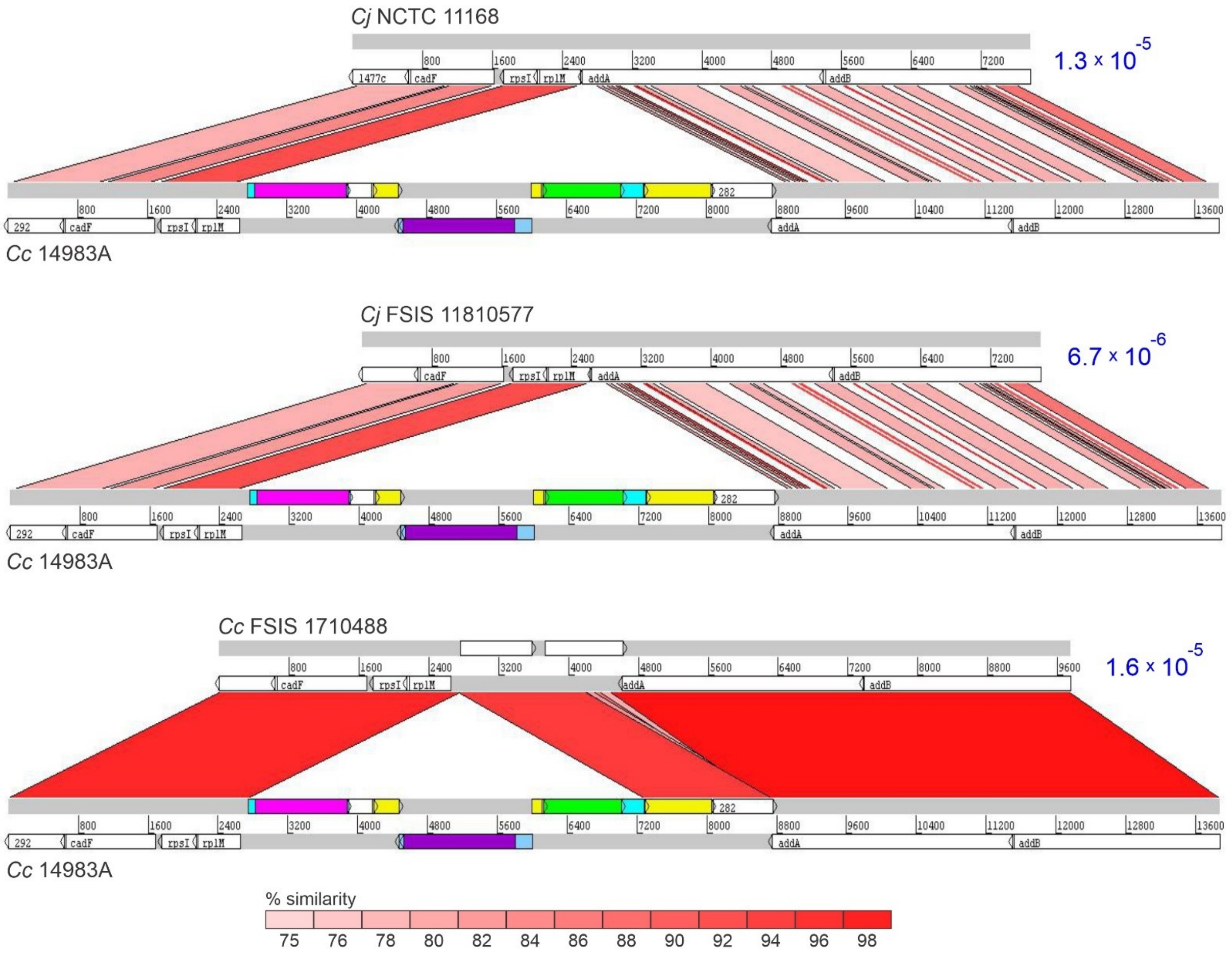

Supplementary Figure S1. Context/similarity comparisons between the genomic regions flanking the gentamicin resistance-encoding element in the donor strain *C. coli* 14983A and the corresponding regions in the three recipient strains *C. jejuni* NCTC 11168, *C. jejuni* FSIS 11810577 and *C. coli* FSIS 1710488. The IS*1595* and IS*605* elements are colored light blue and medium blue, respectively, with their cognate transposase genes colored magenta and violet, respectively. The *aph(2")-If* gentamicin resistance gene is in green. Pseudogenes are yellow. Average transformation frequencies for each pair are shown in blue font on the right. Regions of similarity between each genome pair are colored in shades of red that represent % nucleotide similarity, as determined through BLASTN analysis of the donor against each recipient.

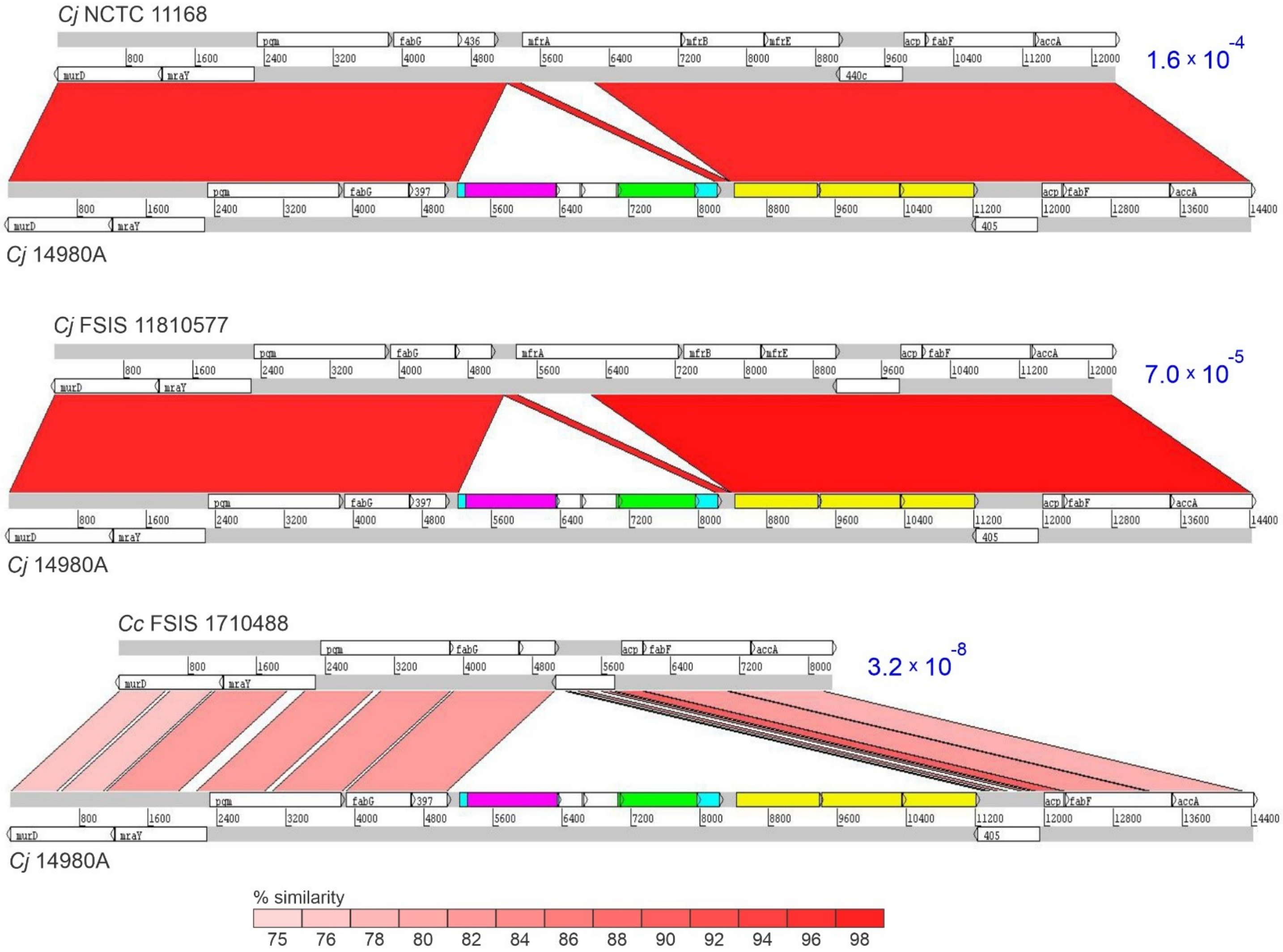

Supplementary Figure S2. Context/similarity comparisons between the genomic regions flanking the gentamicin resistance-encoding element in the donor strain *C. jejuni* 14980A and the corresponding regions in the three recipient strains *C. jejuni* NCTC 11168, *C. jejuni* FSIS 11810577 and *C. coli* FSIS 1710488. The IS*1595* element is colored light blue with its cognate transposase gene colored magenta. The *aph(2")-If* gentamicin resistance gene is in green. Pseudogenes are yellow. Average transformation frequencies for each pair are shown in blue font on the right. Regions of similarity between each genome pair are colored in shades of red that represent % nucleotide similarity, as determined through BLASTN analysis of the donor against each recipient.

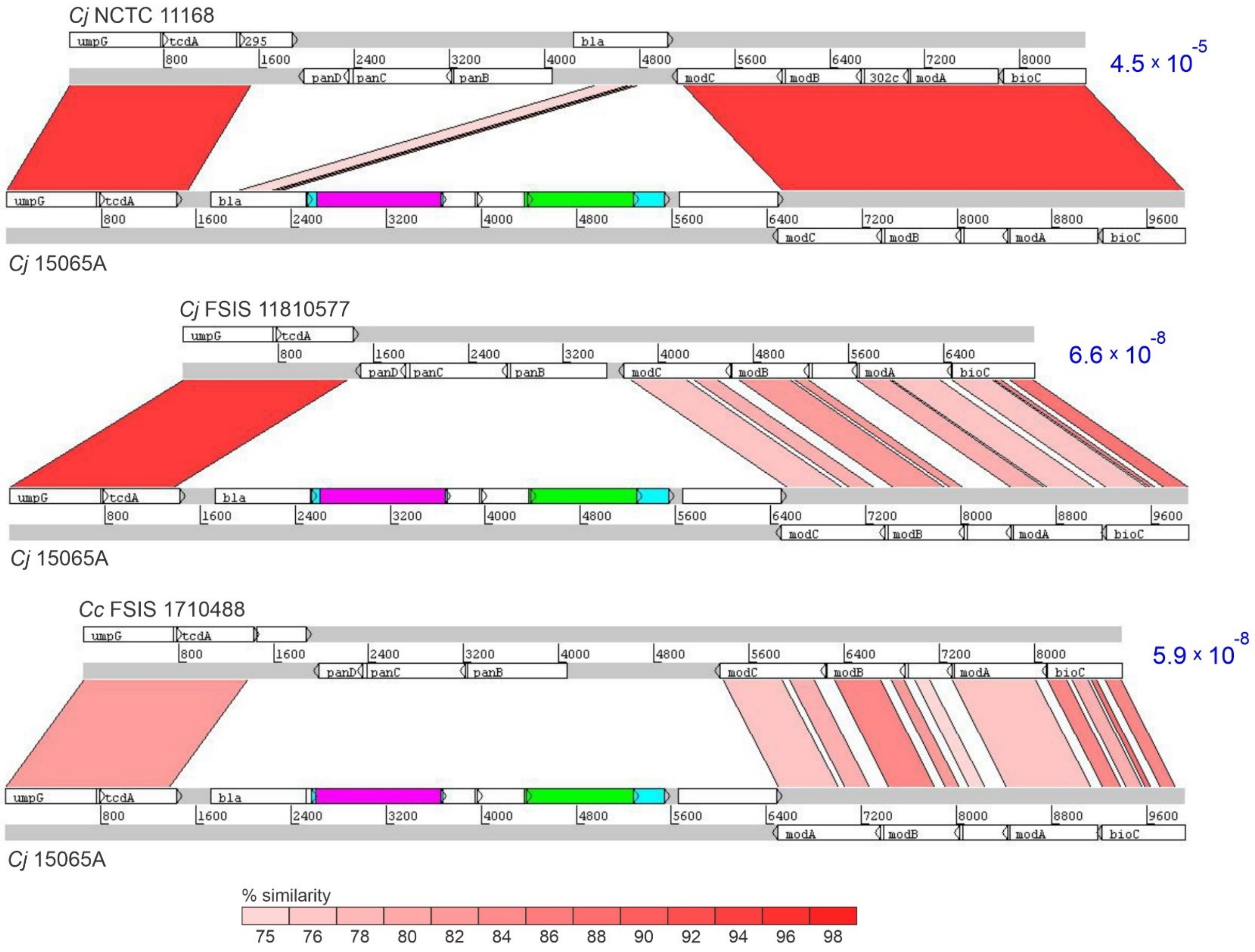

Supplementary Figure S3. Context/similarity comparisons between the genomic regions flanking the gentamicin resistance-encoding element in the donor strain *C. jejuni* 15065A and the corresponding regions in the three recipient strains *C. jejuni* NCTC 11168, *C. jejuni* FSIS 11810577 and *C. coli* FSIS 1710488. The IS*1595* element is colored light blue with its cognate transposase gene colored magenta. The *aph(2")-If* gentamicin resistance gene is in green. Average transformation frequencies for each pair are shown in blue font on the right. Regions of similarity between each genome pair are colored in shades of red that represent % nucleotide similarity, as determined through BLASTN analysis of the donor against each recipient.
